# Muscle Injury Induces Postoperative Cognitive Dysfunction

**DOI:** 10.1038/s41598-020-59639-3

**Published:** 2020-02-17

**Authors:** Lorna Guéniot, Victoria Lepere, Gabriela Ferreira De Medeiros, Anne Danckaert, Patricia Flamant, Marine Le Dudal, Olivier Langeron, Pierre L. Goossens, Fabrice Chrétien, Grégory Jouvion

**Affiliations:** 1 Institut Pasteur, Experimental Neuropathology Unit, Paris, France; 2 Direction Générale de l’Armement, Ministère des Armées, Paris, France; 3 Paris Descartes University, ED Bio-SPC, Sorbonne Paris Cité, Paris, France; 4 Multidisciplinary Intensive Care Unit, Department of Anesthesiology and Critical Care, La Pitié-Salpêtrière Hospital, Assistance Publique-Hôpitaux de Paris, Sorbonne Université, Paris, France; 5 Polyvalent Surgical Resuscitation, La Pitié-Salpêtrière Hospital, Assistance Publique-Hôpitaux de Paris, Sorbonne University, Paris, France; 6 Institut Pasteur, UtechS Photonic BioImaging (Imagopole), C2RT Paris, France; 7 Department of Anesthesia and Critical Care, Hôpitaux Universitaires Henri Mondor-Créteil-Assistance Publique Hôpitaux de Paris, Paris-Est Créteil University, Créteil, France; 8 Service de Neuropathologie, Centre Hospitalier Sainte Anne, GHU Paris Psychiatrie Neuroscience, Paris, France; 9 Sorbonne Université, INSERM, Pathophysiology of pediatric genetic diseases, Assistance Publique-Hôpitaux de Paris, Hôpital Armand-Trousseau, UF Génétique moléculaire, Paris, France

**Keywords:** Neuroimmunology, Neurological disorders

## Abstract

Postoperative cognitive dysfunction (POCD) is a major complication affecting patients of any age undergoing surgery. This syndrome impacts everyday life up to months after hospital discharge, and its pathophysiology still remains unclear. Translational research focusing on POCD is based on a wide variety of rodent models, such as the murine tibial fracture, whose severity can limit mouse locomotion and proper behavioral assessment. Besides, influence of skeletal muscle injury, a lesion encountered in a wide range of surgeries, has not been explored in POCD occurrence. We propose a physical model of muscle injury in CX3CR1^GFP/+^ mice (displaying green fluorescent microglial cells) to study POCD, with morphological, behavioral and molecular approaches. We highlighted: alteration of short- and long-term memory after muscle regeneration, wide microglial reactivity in the brain, including hippocampus area, 24 hours after muscle injury, and an alteration of central brain derived neurotrophic factor (BDNF) and nerve growth factor (NGF) balance, 28 days after muscle injury. Our results suggest for the first time that muscle injury can have early as well as late impacts on the brain. Our CX3CR1^GFP/+^ model can also facilitate microglial investigation, more specifically their pivotal role in neuroinflammation and synaptic plasticity, in the pathophysiology of POCD.

## Introduction

Postoperative cognitive dysfunction (POCD) is a well-recognized phenomenon affecting patients of any age undergoing surgical procedures^1,2^. This cognitive decline can be observed among 5 to 40% of patients^3^, lasting few days to several months after surgery^4^. It impacts numerous cognitive abilities such as attention, speed of information processing, executive function, ability to combine tasks, psychomotor dexterity and memory^5,6^. These dysfunctions are factors of morbidity, impairment of everyday life, and even mortality, with a strong impact on global health-care burden^7,8^.

Despite long-term awareness of this phenomenon^9^, multifactorial pathogenesis of POCD still remains unclear. Only aging has been confirmed as an important susceptibility factor^2^. A wide variety of surgeries may lead to POCD, but the type of surgery and anesthesia does not appear to influence its incidence in non-cardiac surgeries^10^. Among older patients, type of hospitalization appears to have a role as out-patient approach seems to lower POCD occurrence after minor surgery^11^. Recent experimental studies are bringing new perspectives on the subject: systemic inflammation and chronic pain have been suggested as the major actors involved in cognitive impairments by modulating neurotrophic factors in the hippocampus, a central structure in memory formation^12–15^. This modulation could involve microglial cells^16^, that play a key role in neuroimmunomodulation^17^ and synaptic plasticity^18^. Alteration of their functions by aging has already been incriminated in age susceptibility to POCD^19,20^.

In experimental research, murine tibial fracture is one of the most widely used animal model to mimic POCD^15,21,22^. It combines sufficient tissue destruction and chronic pain to allow neurologic impairments even in young adult mice, while other models of surgery-induced cognitive decline - such as splenectomy^23^ and laparotomy^24,25^ - need to be executed in more susceptible mice (older or females). However the longer period of its histological and functional recovery (>4 weeks^26^) can limit the proper highlighting of POCD during the first month after surgery, as most of murine behavioral assessments require spontaneous locomotion without any sensitivity or mechanical bias.

Our aim was to propose a less traumatic murine model of POCD with a faster recovery period after surgical procedure. It allowed observation of cognitive impairment while histological and functional recovery of limb was complete, even in young adult male mice. For this purpose, we used freeze-injury (FI) of the *Tibialis anterior* muscle. Despite being a widely used model for muscle injury induction, its effect on the central nervous system (CNS), and neurocognitive functions in particular, has not been described yet^27^. As muscle destruction (in traumas or surgeries) is a very common insult, its influence on POCD occurrence thus needs to be addressed. In this study, early morphological reactivity of microglia, late cognitive function and brain neurotrophic levels were precisely assessed after muscle surgery.

## Materials and Methods

### Animals

This study was performed in accordance to French and EU guidelines for animal care. All protocols were approved by the Ethics Committee of the Institut Pasteur and the French Ministry of Research (Ref: APAFIS#9210-2017031014524355v3). In-house CX3CR1^GFP/+^ male mice aged from 6 to 8 weeks at lesion induction were used for experiments. Mice were housed in cages in groups of five or six, monitored every day, with food and water *ad libitum*, in a temperature (22 ± 1.5 °C) and humidity-controlled environment with a 12 h light/dark cycle. Our experiments had a limited impact on the mouse living condition. Our endpoints were in accordance to the welfare score grid of the Institut Pasteur (Table 1); no mouse reached any of these endpoints during our experiments.

**Table 1:** Welfare score grid of Institut Pasteur (Paris).

Parameter	Level	Score
*Appearance*	Normal	0
	Ruffled coat, moderate facial expression, move	1
	Hunched back, severe facial expression, do not move	2
*Cachexia Body Condition Score*	BCS 3	0
	BCS 2	1
	BCS 1	2
*Body weight*	Normal (≥initial weight)	0
	Weight loss <20% initial weight	1
	Weight loss >20% initial weight	2
*Specific endpoints*	No lameness	0
	Moderate lameness	1
	Severe lameness	2
**Total**		**/8**

Monitoring welfare grid of Institut Pasteur (Paris) with qualitative and quantitative criteria. The best welfare is leading to a score of 0/8, the worst 8/8. For a score equal or above 2/8, euthanasia is mandatory.

### Muscle freeze injury procedure

Surgical muscle destruction procedure with no exogenous substance susceptible to have a direct effect on the CNS was chosen. As previously described^28^, mice were anesthetized with 4% isoflurane delivered in 1.5 L/min air and maintained with 2% isoflurane. Correct analgesia was achieved with preoperative buprenorphine (0.3 mg/kg i.p.). For each mouse, the left calf skin was incised on 0.5 cm next to the *Tibialis anterior* (TA) muscle for expositing it (Sham and Freeze-injured (FI)). The TA was frozen with three consecutive cycles of freeze-thawing by applying for 15 s a liquid nitrogen-cooled metallic rod only for FI mice. The skin was then sutured and animals kept at 37 °C on a heating pad until waking up. In every experiment, Sham mice (anesthesia + analgesia + skin incision + suture without TA freezing) were used as control for highlighting the influence of muscle injury.

### Time points and number of animals in each group

Following surgery, mouse euthanasia was carried out at several time points: (i) 24 hours post-injury to describe early alterations (completed by 2 time points 3 and 5 days post-injury, for TA muscle histopathological analysis) and (ii) 28 days post-injury for the evaluation of long-term consequences. At early time points (1, 3 and 5 days post-surgery), 5 mice were used for Sham group and 6 for FI group. At late time point (28 days), 11 mice were used for Sham group and 10 for FI group. A repetition with an equivalent number of mice was conduct to confirm statistically significant results. Removal of mice due to technical considerations is described in 2.8.

### Behavioral studies

The same cohort of animals was subjected to the behavioral tests described below to explore their cognitive function, especially memorization process implicating hippocampal area. All behavioral evaluations were performed after muscle regeneration and locomotor recovery (during the 3^rd^ week after surgery). All behavioral tests took place during the light phase of the light/dark cycle. Each quantification was performed on video by a blind trained experimenter.

#### Open field

On the 21^st^ day after surgery, mice were submitted to the open field. Mice were individually placed inside the open field arena and left to explore it for 5 minutes. Light was ≈100lux in the center, ≈50lux close to the walls. The total distance moved, time spent in the bright area and number of fecal pellets were quantified. A reduced locomotion can suggest locomotor impairment or apathy, or an anxious phenotype when restricted to the darker area of the apparatus^29^.

#### Novel object recognition (NOR)

This test was performed to assess memory function^30^, the day after open field evaluation. Briefly, mice were first placed into an open field arena containing two identical objects (randomly two lab glass bottles or two ceramic jars) until they reached a criterion of 30 s of total exploration for both objects (training session). Exploration time was registered when the snout of the mouse was directed towards the objects from a distance shorter than 2 cm (climbing was excluded). Long-term memory was evaluated first during the test session performed 24 h after the training session (24 h NOR). Mice were placed in the same arena with one of the familiar objects randomly replaced by a novel one. The time exploring these objects was again quantified until a criterion of 30 s of total exploration was reached (cutoff of 5 minutes). Short-term memory was evaluated 3 h later with a novel test session (3 h NOR), introducing a third new object (a funnel) and time exploring was recorded the same way as 24 h NOR test session. In the two test sessions, preference for the novel object was evaluated though a ratio between the time spent exploring the novel object and the total (familiar + novel) exploration time. In this test, success is defined for a group by a score significantly above the chance level (50%)^28^. Short and long-term memories were evaluated for the same mice in the control and FI groups.

#### Y-maze

After NOR evaluation, spatial memory was assessed with the tendency for mice to alternate their exploration choices of Y-maze arms^31^. A mouse was placed in the center of the apparatus and the number and order of “full entries” (beyond the first quarter) of each arm (labeled A, B or C) was registered during 5 minutes. The number of correct alternations (ABC, ACB, BCA, BAC, CBA, CAB) was evaluated in every triad of entries. Then, a ratio was made between the number of correct alternations and the total number of possible alternations (number of total entries less 2). A number of alternation greater than chance level (22.2%) reflects the ability of the mouse to remember the previously entered arms and hence to alternate its choices. The total number of entries was also used as a locomotion index.

### Tissue preparations

Mice received lethal anesthesia with ketamine-xylazine (30 mg/kg and 150 mg/kg i.p., respectively) at different time points.

#### Tibialis anterior muscle analysis

The left *Tibialis anterior* (TA) muscles were collected 24 hours, 3 days, 5 days or 28 days after cryolesion (to ensure that the muscle was (i) necrotic after the cryolesion and (ii) regenerated 28 days post-injury) and snap-frozen in liquid nitrogen-cooled isopentane. At least four different levels of 7 µm-thick sections of each TA muscle were cut and stained with hematoxylin-eosin (HE).

#### Brain analysis

Brains were removed 24 hours or 28 days post-muscle injury, after intracardiac perfusion of cold NaCl 0.9% (20 mL) and cut in a trans-sagittal plane in the hemispheric fissure. Left cerebral hemispheres were immediately frozen in liquid nitrogen. Right cerebral hemispheres were fixed for 24 h in 10% neutral-buffered formalin at room temperature.

### GFP image acquisition and analysis

For precise and quantitative assessment of microglia morphology, we adapted our previously described protocol using CX3CR1^GFP/+^ mice^32^. Briefly, right cerebral hemispheres were sliced along a sagittal plane on a calibrated vibratome into 100 µm-thick free-floating slices. Using a spinning disc confocal system (CellVoyager CV1000, Yokogawa, Japan) with a UPLSAPO 40×/NA 0.9 objective, sample areas were acquired as a square of 5 × 5 fields (920 × 920 pixels/field, pixel size in X and Y dimensions was 0.19 µm according to the objective) of view with a depth of 30 µm at 2 µm increments (16 focal depths) generating one volume in 7 regions of interest: olfactive area (OA), frontal cortex (FC), cerebral nuclei (CN), hippocampus (HP), thalamus (TH), hypothalamus (HT), and midbrain (MB). The 488 nm laser was used to excite GFP. Focal stacks of each mosaic were reconstructed by combining images from the different focal depths using automated free plugins^33^ of ImageJ v1.50 software interface^34^. Using our custom-designed script^32^ developed with the Acapella^TM^ image analysis software (version 2.7, PerkinElmer Technologies, Waltham, USA), we extracted the following morphologic criteria for each GFP-positive cell: cytoplasmic area (defined as the area of the cytoplasm included in the primary branches) and cell environment area (represented by the 2D total surface covered by ramifications, and defined as the area of the polygon formed by linking the extremities of microglial processes) expressed in μm^2^. The complexity score (CS) was defined by the ratio between the number of segments of each ramification of each cell, multiplied by the sum of the nodes on one hand and the number of primary branches on the other hand. The CS was calculated according to the following formula:

$$CS=\frac{nb\,of\,segments\times (nb\,of\,nodes1+nb\,of\,nodes2)}{nb\,of\,roots}$$

At least 100 microglial cells were automatically selected by our custom-designed script and analyzed for each brain region and each mouse. Image intensity was measured by Acapella^TM^ software and controlled as a cofounding factor between the two groups of mice.

### Neurotrophin analysis

Mice were sacrificed at least two days after the end of behavioral studies, to avoid the highlighting of transitory variation in biomarkers. Frozen left cerebral hemispheres were weighed (for normalization) and disrupted with 200 µL/400 mg of extraction buffer (PBS 1x and cOmplete^TM^ EDTA-free protease inhibitor, Roche^®^, Mannheim, Germany) in 2 mL Lysing Matrix Z tubes (MP Biomedicals^®^, Germany) with FastPrep-24 homogenization system. Then, tubes were centrifuged at 10,000 rpm at 4 °C during 10 min. Brain homogenates were collected either for Luminex^®^ flow cytometry technique (Magnetic Luminex Screening Assays, LXSAMSM-13, R&D Systems^®^) on DropArray^TM^ microplate (Curiox Biosystems^®^, Singapore) for nerve growth factor (β-NGF) assay; or for enzyme-linked immunosorbent assay (ELISA kit, DBNT00, R&D Systems^®^, Minneapolis, MN, USA) for brain neurotrophic factor (BDNF). Protocols were performed according to manufacturer’s instructions.

### Data analysis and statistics

Statistical analysis was performed with Graph-Pad-Prism software version 6.0 (GraphPad Software Inc.^©^, La Jolla, CA). For microglial morphology, outlier cells only were removed using ROUT GraphPad method per region for each morphologic criterion. Only remaining microglial cells with CS > 1 were used for morphological comparison. In the Open field test, one Sham mouse was removed before analysis because of a video recording error, as another Sham mouse in Y-maze test. In the 3 h and 24 h NOR test, two Sham mice have been removed before analysis: one for a strong side preference during the training session with two identical objects, and one for lesions in its left eye. In the 3 h NOR, one Sham and one FI mice have been removed before analysis because of a placement error regarding the side of the novel object (non-respect of the random selection). In NOR and Y-maze tests, group median were compared to their chance levels (50% and 22.2%, respectively) with Wilcoxon Signed Rank test for median comparison. Rest of the data were analyzed using the Mann-Whitney test after being assessed for non-normal distribution, and represented by median (med) and interquartile range (IQR). Statistical significance was taken at p < 0.05. A possible limitation of this statistical analysis is the potential increase in Type I error, considering the total number of statistical tests performed within a single set of analyses. For neurotrophin analysis, all experiment repetitions were pooled to increased statistical significance of BDNF analysis; Five frozen brains of each group (Sham and FI) were lost in an accidental thawing.

## Results

### Freeze-injury of *Tibialis anterior* muscle as a mild surgical procedure with a quick recovery period in young adult male CX3CR1^GFP/+^ mice

Freeze injury (FI) of *Tibialis anterior* (TA) muscle is a widely used model of physical muscle injury, with no diffusion of exogenous substance and complete regeneration within 1 month^28^.

#### FI of TA muscle is an effective muscle injury with a complete regeneration in 3 weeks

**Figure 1:**
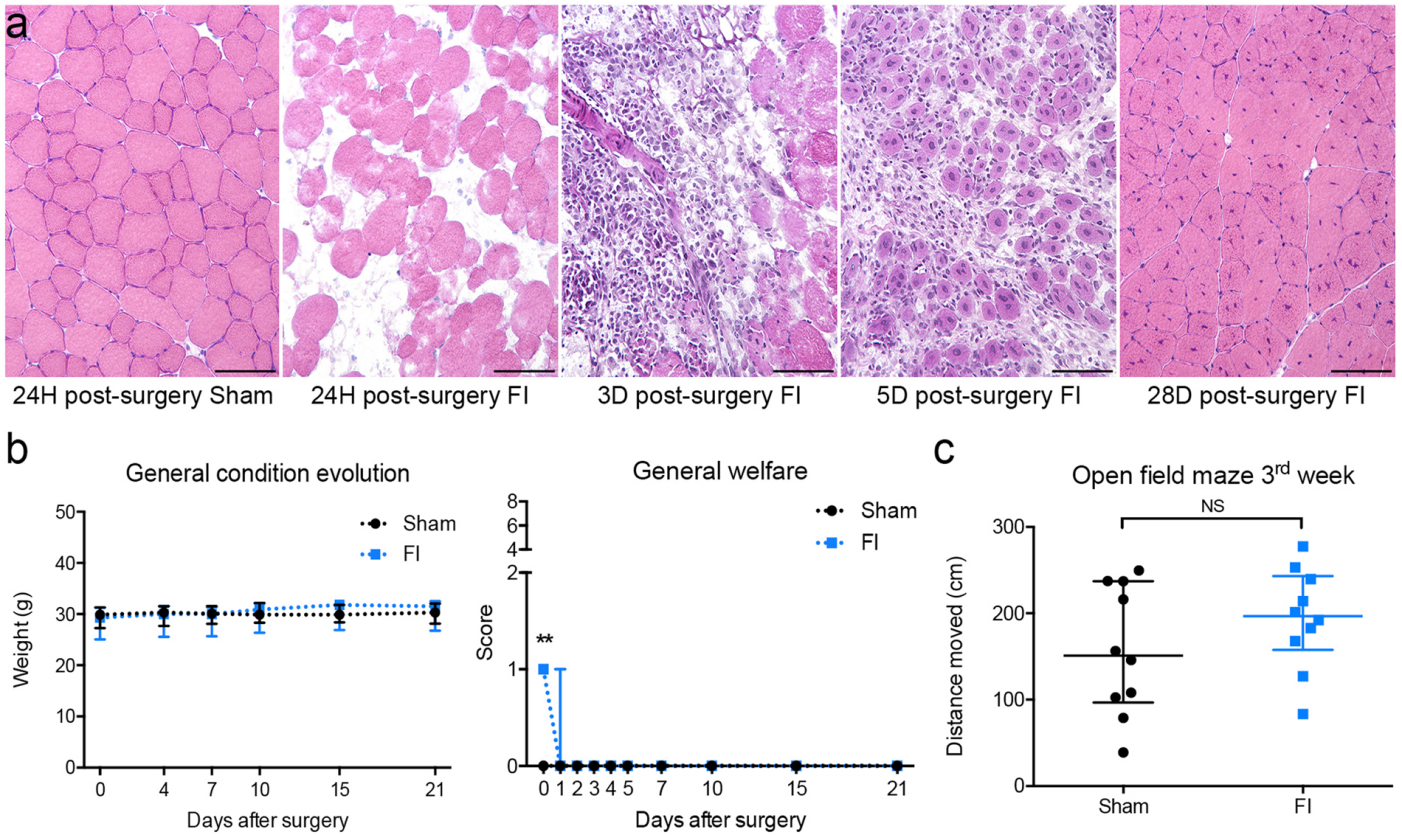
Muscle freeze-injury induces only mild general disturbance and muscle regeneration within 3 weeks in young adult male CX3CR1^GFP/+^ mice. (**a**) Hematoxylin-Eosin staining on cryosections. Sham mice did not display any skeletal muscle lesion in these procedures. In contrast, muscle freeze injury (FI) induced necrosis of muscle tissue (24 h post-injury), and regeneration (completed 28 days post-injury, with the remaining of central nuclei). Scale bar = 100 µm. (**b**) General impact of FI was characterized by weight preservation within 3 weeks after the procedure, and by moderate lameness observed for all FI mice in the first 24 hours among criteria of welfare score (Table 1) resulting in a score at 1/8. All Sham mice get the best welfare score (0/8), during the entire protocol period (n = 11 for Sham group, n = 10 for FI group). (**c**) Muscle functional recovery was characterized by similar locomotion between FI and Sham group in open field evaluation, 21 days after the surgery (n = 10 for each group). Data are represented as median with inter-quartile range. Significance is indicated with asterisk (**p < 0.01, NS: non-significant); analyzed with Mann-Whitney U test.

In young adult CX3CR1^GFP/+^ male mice, extending our previous model in C57BL6/J mice^28^, the FI procedure induced (i) necrosis of muscle tissue 24 hours post-surgery and freeze-injury and (ii) regeneration (with the remaining of central nuclei, a common phenomenon in the mouse^28^) 28 days post-injury. In contrast, Sham mice did not display any skeletal muscle lesion (Fig. 1a) except small inflammatory mononuclear cell infiltrations in the epimysium during the first 24 hours and rare central nuclei at 28 days (data not shown).

#### FI of TA muscle has a low clinical impact

General welfare was evaluated using the mandatory score of our ethical committee in Table 1. Only a moderate hindlimb lameness was observed on the 1^st^ day after muscle injury for the FI group, resulting in a welfare score at 1/8 for all the FI mice without abnormal facial expression or ruffled coat. On the 2^nd^ day, some mice of the FI group still presented a moderate lameness. From the 3^rd^ day, no lameness was observed for all the FI mice and welfare score was similar to those of Sham. All Sham mice get the best welfare score (0/8), during the entire protocol period (Fig. 1b). Weights remained stable and similar between the two groups during the first 3 weeks after the procedure (Fig. 1b).

#### FI of TA muscle allows a similar locomotion between Sham and FI mice 3 weeks after surgery

Functional muscle recovery was assessed when histological regeneration of TA muscle is quite achieved at 21 days after surgery^28^. The open field test is a popular evaluation of spontaneous locomotion in mice^29^. No difference either in distance moved or velocity was seen between Sham and FI groups during the 5 min testing (Fig. 1c).

Taken together, these results suggest that FI of TA is a mild surgical procedure with a quick recovery period.

### Early microglial reactivity 24 hours after muscle lesion

To characterize a potential early impact of the muscle injury on the central nervous system (CNS), microglia morphology was evaluated 24 h after surgery. Microglial cells are tissue-resident “macrophages” of the CNS with highly dynamic morphology, physiologically sensitive to their microenvironment^35^. We used the automated high-content analysis tool developed by our team for characterizing their morphology^32^.

**Figure 2:**
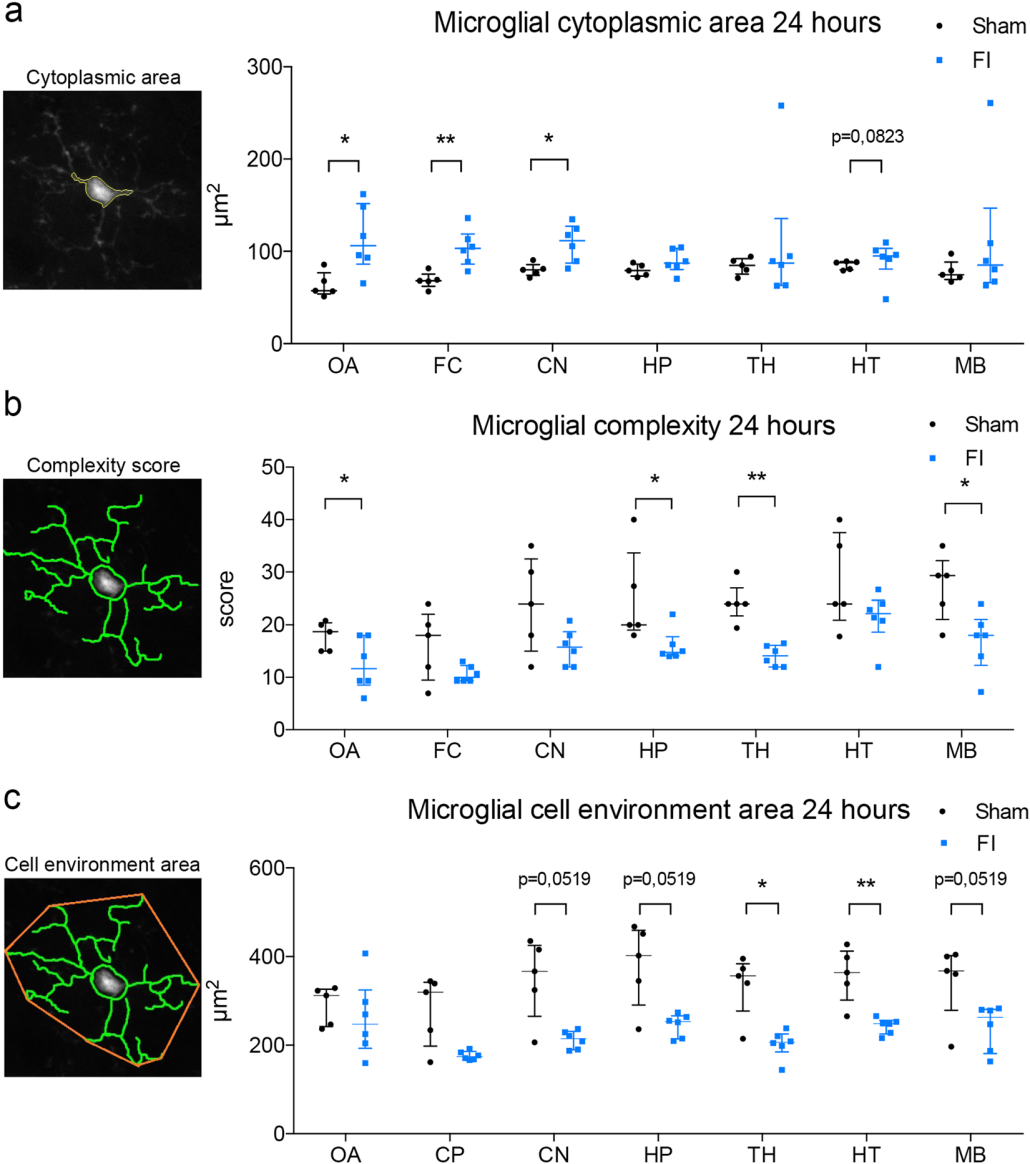
Freeze-injury induced widespread microglial morphological changes in brain 24 hours after surgery in young adult male CX3CR1^GFP/+^ mice. Automated morphometric analysis of microglial cells in olfactive area (OA), frontal cortex (FC), central nuclei (CN), hippocampus (HP), thalamus (TH), hypothalamus (HP) and midbrain (MB). (**a**) Microglial cytoplasmic area was significantly increased in OA, FC and CN for FI group compared to Sham. The same tendency was observed in HT. (**b**) Microglial complexity score, reflecting the ramification of microglial cells, was significantly decreased in OA, HP, TH and MB for FI group compared to Sham. (**c**) Microglial cell environment area was significantly decreased in TH and HT for FI group compared to Sham. The same tendency was observed in CN, HP and MB. (n = 5 for Sham group, n = 6 for FI group). Data are represented as median with inter-quartile range. Significance is indicated with asterisk (*p < 0.05, **p < 0.01); analyzed with Mann-Whitney U test.

Significant microglial morphological changes were observed in the 7 encephalic regions analyzed 24 h after surgery, in FI group compared to Sham (Fig. 2). The cytoplasmic area was indeed increased in FI mice for olfactory area (p = 0.0173), frontal cortex (p = 0.0087) and central nuclei (p = 0.0173) compared to Sham. The same tendency was observed for hypothalamus (p = 0.0519) (Fig. 2a).

Moreover, the microglial complexity score was decreased in olfactive area (p = 0.0476), hippocampus (p = 0.0260), thalamus (p = 0.0043) and midbrain (p = 0.0455) in FI group compared to Sham (Fig. 2b).

Furthermore, the environment area covered by microglial cells was decreased in FI mice for thalamus (p = 0.0173) and hypothalamus (p = 0.0087); the same tendency was also observed in central nuclei (p = 0.0519), hippocampus (p = 0.0519) and midbrain (p = 0.0519) compared to Sham (Fig. 2c).

These results are consistent with a decrease of complexity of microglial cytoplasmic processes; microglial cells adopt a more amoeboid form (phagocytic and migratory, in contrast to quiescent ramified microglia). This microglial reactivity was global and occurred within 24 h after muscle surgery. It involved hippocampus and FI group presented a more active morphological profile.

### Altered cognitive abilities 3 weeks after muscle lesion

As muscle recovery was completed 3 weeks after the surgical procedure, we assess the long-term clinical consequences of such a peripheral injury on the CNS through cognitive evaluation. Unlike studies that use fear conditioning with electric stimulation to evaluate memory^15,22^, we chose spatial and novel object recognition (NOR) based on the spontaneous behavior of mice avoiding a potential bias with differential limb sensitivity due to peripheral injury^15^, even when recovery is achieved^36^.

#### FI of TA muscle induces a similar level of anxiety between Sham and FI mice 3 weeks after surgery

**Figure 3:**
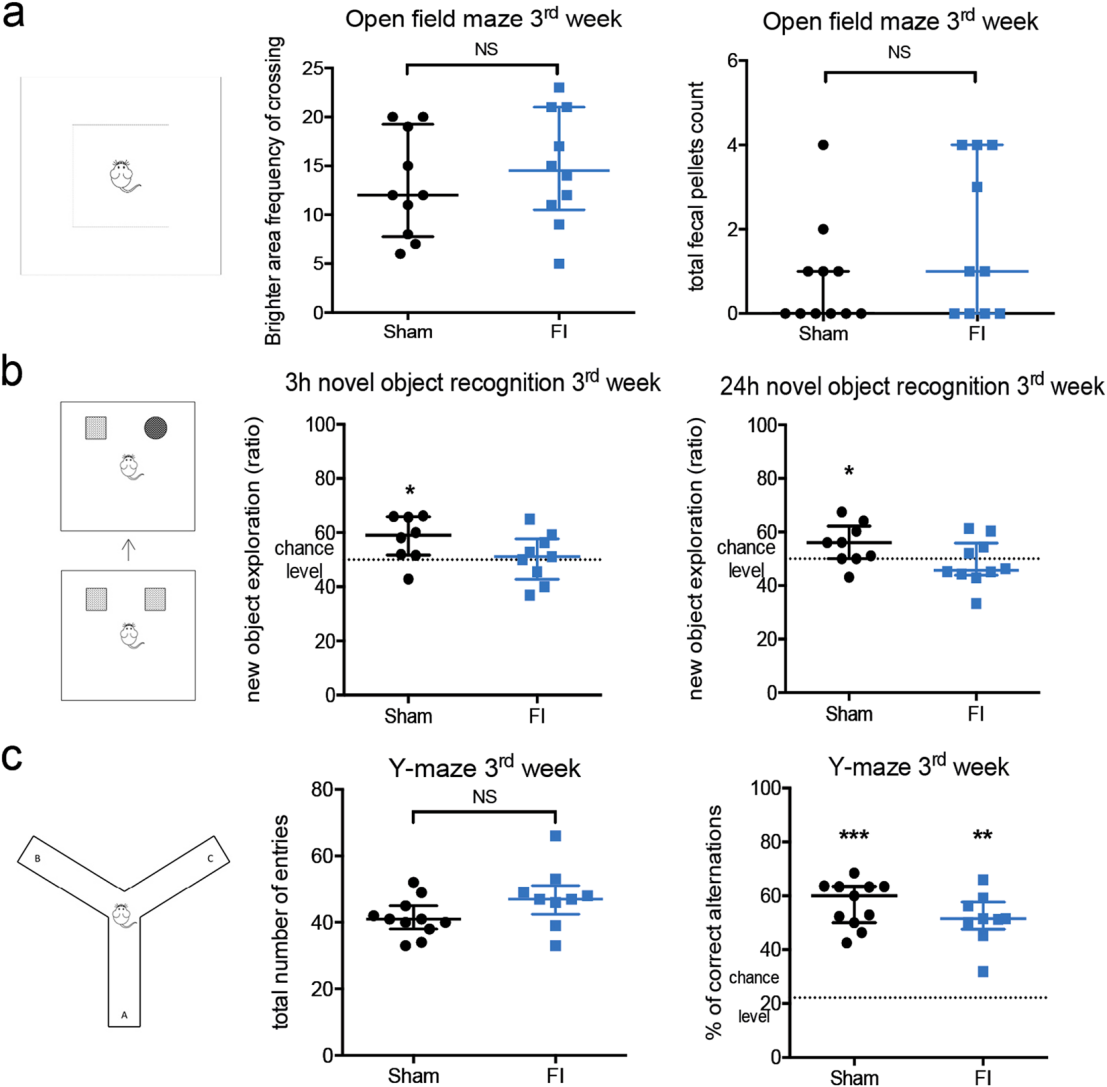
Freeze-injury induced cognitive impairments 3 weeks after surgery in young adult CX3CR1^GFP/+^ mice. (**a**) Before evaluating memory process in both groups, level of anxiety was controlled during open field maze test to avoid any interference in the following memory evaluations. No differential anxiety was revealed between Sham and FI groups (n = 10 for Sham group in crossing frequency of brighter area and 11 in total fecal pellet count, n = 10 for FI group). (**b**) Novel object recognition (NOR) test was used to assess either short- and long-term memory. Success in this test is defined by a group score above the level chance (50%). FI group failed in the memory evaluation either at 3 hours (med 51% [IQR 43–58] *p* = 0.8070) or 24 hours (med 46% [IQR 41–60] *p* = 0.9821), in contrast to Sham group (3 h NOR: med 59% [IQR 52–66] *p* = 0.0391, n = 8 for Sham group and n = 9 for FI group; 24 h NOR: med 56% [IQR 49–61] p = 0.0195, n = 9 for Sham group and n = 10 for FI group). (**c**) To investigate another type of memorization process, immediate spatial memory was evaluated using spontaneous alternation test (Y-maze test). Total number of entries in all arms was similar in Sham and FI mice. Both groups succeeded as they were significantly above the chance level of 20% (n = 11 for Sham group, n = 9 for FI group). Data are represented as median with inter-quartile range. Significance is indicated with asterisk (*p < 0.05, **p < 0.01, ***p < 0.001); analyzed with Wilcoxon Signed Rank test for median comparison with 50% and 22.2%, analyzed with Mann-Whitney U test for group comparison. Mouse drawing by Clker-Free-Vector-Images from Pixabay.

Spontaneous participation of mice in memory testing requires a similar level of anxiety among the different groups, so that its potential interference in cognition does not stand as a confounding factor. Anxious behavior in FI and Sham mice was assessed using the same open field test as in §*3.1*. In this evaluation, avoiding the brighter central area and spending more time close to the walls were used as indexes of anxious behavior^37^. Defecation during the test is also used as a semi-quantitative measure of stress. The level of anxiety was similar between sham and FI mice, either in term of time spent in the different areas or fecal pellet count (Fig. 3a).

#### FI of TA muscle alters short and long-term memory 3 weeks after surgery

Novel object recognition test was used to assess both short and long-term memory.

In the 3 h NOR assessing short-term memory, the Sham group explored the novel object above chance level (med 59% [IQR 52–66] p = 0.0391) confirming the short-term recognition of the new object (Fig. 3b). The FI group, on the other hand, explored both the familiar and the novel object evenly (med 51% [IQR 43–58] p = 0.8070), suggesting a deficit in the recognition of the novel object (Fig. 3b).

An alteration of long-term memory of FI mice was also observed. The Sham group indeed succeeded in the recognition of the new object 24 h after the training session (med 56% [IQR 49–61] p = 0.0195) while the FI group did not (med 46% [IQR 41–60] p = 0.9821) (Fig. 3b).

#### FI of TA muscle preserves immediate spatial memory 3 weeks after surgery

We evaluated immediate spatial memory using the spontaneous alternation test (Y-maze). The use of random criteria in the arm choice would result in a 22.2% percent of correct alternations, and hence a higher percentage of correct alternations implies a greater immediate spatial memory retention^31^. In this test, exploration levels were similar between Sham and FI mice (Fig. 3c). Both groups succeeded in this test (Sham: med 60% [IQR 50–63] p = 0.0010; FI: med 52% [IQR 48–58] p = 0.0039) (Fig. 3c).

Taken together these results demonstrate a late alteration of cognitive abilities in mice submitted to FI (short-term and long-term memories), despite complete muscular regeneration.

### Brain levels of neurotrophins 3 weeks after muscle lesion

The late cognitive impairments involving memorization process lead us to evaluate brain levels of two major central neurotrophic factors: brain derived neurotrophic factor (BDNF) and nerve growth factor (NGF). These two neurotrophins play an important role in neuronal function and synaptic plasticity^38,39^ and their potential implication in POCD has recently been described^13,15^.

**Figure 4:**
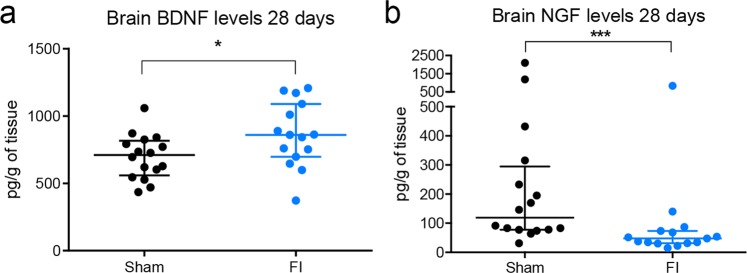
Freeze-injury altered brain levels of neurotrophins 3 weeks after surgery in young adult CX3CR1^GFP/+^ mice. On the same animal cohort presenting differential cognitive performances, levels of brain derived neurotrophic factor (BDNF) and nerve growth factor (NGF) were measured in brain homogenate 28 days after surgical procedure. (**a**) Brain levels of BDNF were significantly increased in FI mice (med 860 pg/g of tissue [IQR 700–1090]) compared to Sham (med 710 pg/g of tissue [IQR 560–820]; p = 0.0296) in ELISA assay. (**b**) Brain levels of NGF were significantly decreased in FI mice (med 48 pg/mL [IQR 31–74]) compared to Sham (med 119 pg/mL [77–295]; p = 0.0009) in multiplex assay. Data are represented as median with inter-quartile range (n = 15 for each group). Significance is indicated with asterisk (*p < 0.05, ***p < 0.001); analyzed with Mann-Whitney U test.

After completing the behavioral assessment, central levels of neurotrophic factors were evaluated on the same mice cohort 28 days after the surgery. Brain BDNF levels were significantly different between the two groups, with an increase observed in FI mice compared to Sham (FI: med 860 pg/g of tissue [IQR 700–1090]; Sham: med 710 pg/g of tissue [IQR 560–820]; p = 0.0296) (Fig. 4a).

Moreover, a strong decrease of NGF was revealed in the brain of FI mice compared to Sham (FI: med 48 pg/mL [IQR 31–74]; Sham: med 119 pg/mL [77–295]; p = 0.0009) (Fig. 4b).

Thereby, cognitive impairments after muscle regeneration were associated with an increase of brain BDNF level and a decrease of brain NGF level.

## Discussion

Translational research is trying to decipher the underlying mechanisms leading to cognitive dysfunction after surgery (POCD), progressively incriminating tissue destruction as a triggering factor. For this purpose, murine tibial fracture with intramedullary pinning is a widely used model allowing observation of POCD even in young adult mice (12–14 weeks). Nevertheless, the recovery period for mouse limb is superior to 4 weeks^26^, susceptible to interfere with behavioral evaluation carried out during this period. To our knowledge, our study is the first to evidence POCD in a model of muscle injury, in young adult male mice (12–14 weeks), when histological recovery from surgery is complete. We highlighted memory alterations involving short- and long-term object recognition memory, 3 weeks after the muscle injury. Moreover, this POCD was demonstrated without any bias coming from differential locomotion of mice, which is highly required for quality of cognitive evaluation. Our model could also shed light on non-surgical muscle injury influence on cognition, as a clinical study revealed a similar impaired cognitive score between athletes, presenting either head concussion or musculoskeletal injuries^40^. Numerous surgeries imply a muscle effraction and if this tissue has a specific role in POCD occurrence, it needs to be addressed.

To our knowledge, we are also the first to highlight a widespread morphological reactivity of microglia in the brain (including hippocampal area) 24 h after muscle surgery. Recent experimental findings suggest an essential role of the brain resident immune system in POCD occurrence. In a murine model, it was demonstrated that depleting microglial cells prevented both hippocampal inflammation and cognitive decline after tibial fracture^21^. Pivotal role of microglia was also shown in a rat model of POCD using minocycline to inhibit microglial activation^41^. Surprisingly in this model, the microglial activation was delayed and worsened the cognitive decline after laparotomy in aged rats. Microglia influence has also been incriminated in age susceptibility to POCD^19,20^.

CX3CR1^GFP/+^ mice have green fluorescent microglial cells (insertion of the GFP reporter gene in one copy of the fractalkine receptor gene); this murine model with surgery-induced cognitive impairments is very helpful to explore microglial cell potential involvement in POCD pathophysiology. Besides, studies using CX3CR1^GFP/+^ mice have not evidenced any deficits in muscle injury repair^42^ nor spontaneous locomotor activity^43^. The impact of disruption of one copy of the fractalkine receptor gene would also be very interesting to study, as it has already been reported differences in synaptic plasticity and memory function, with CX3CR1^GFP/+^ mouse strain^43^. We cannot exclude that this modified genotype could have an impact on neurocognitive deficits (addition of an increase responsiveness towards peripheral insults or masking an even greater severity of the symptoms).

In addition to the POCD observed in our muscle freeze injury procedure, two major neurotrophic factors presented altered levels in brain 28 days after surgery. First, we found an increase of brain derived neurotrophic factor (BDNF) in brain homogenate. This finding is coherent with the rise of BDNF levels both in the dorsal root ganglia and the hippocampus in murine tibial fracture with intramedullary pinning on young adults^15^. In aged patients, decreased serum levels of BDNF seem to correlate with postoperative delirium and POCD occurrence^44,45^ and interestingly, these serum levels also correlate with cerebrospinal fluid concentrations^46^. However, no clinical study specifically addressed the question of brain BDNF variation in the context of POCD. Since we evaluated BDNF levels for the entire brain, the general increase observed could be hiding significant regional differences. As a matter of fact, it has been shown that hippocampus and amygdala do not regulate BDNF pathway in the same way after murine tibial fracture^15^. Also, the tissue assays performed provide information on target presence but not on its bioavailability: microglial or neuronal release of BDNF^47,48^, or deficit in its receptors, could be impaired in our experiments and further investigations will be carried out to better understand these specific questions.

In addition, we observed in the brain a decrease level of nerve growth factor (NGF), which has not been demonstrated previously in rodent models of surgery-induced cognitive decline. NGF is an essential neurotrophin for survival and regulation of basal forebrain cholinergic neuron (BFCN) function. Its decrease was shown to affect BFCN functions, resulting in memory issues in Alzheimer’s disease^38^. Impaired NGF signaling pathway is also described in mild cognitive impairment resulting to aging^38^ or sepsis^23^, and recent therapeutic approaches in secondary cognitive impairments are aiming to restore normal expression or/and level of NGF^49^. We hypothesize that the decrease in brain NGF levels observed in FI mice 28 days after surgery, more than a marker of memory dysfunction, plays an active role in the processes leading to cognitive impairment after surgical intervention. This role might partially rely on the neuroprotective phenotype NGF confers to microglial cells^50^.

Regarding the etiology of cognitive decline appearance in our experiment, several mechanisms need to be explored. First of all, due to its wide morphological reactivity through the brain 24 h after induction of cryolesion, microglia activation needs to be addressed. Either by promoting neuroinflammation^17^ or/and by its participation to synaptic plasticity^18^, microglial cells could have a pivotal role in the acquisition of POCD. Our model can facilitate any microglial exploration, especially to control the repercussion of its regulation by some drugs such as minocycline^51^. If such implication of resident immune system is confirmed, preventive therapeutic strategies can be improved by resolving the “how” and “when” microglia is “aware” of sterile peripheral traumatic insult. Destruction of *Tibialis anterior* muscle can initiate a sufficient inflammatory reaction altering the brain blood barrier (BBB) or diffusing through brain regions without BBB (circumventricular organs) and leading to microglial reactivity. A retrograde pathway in CNS can also be considered as lesions of sciatic nerve lead to wide ipsilateral reaction of microglia in spinal cord, affecting microglia reactivity in brain areas too^52^. Microglia reactivity in spinal dorsal horn has also been shown to play a pivotal role in allodynia mechanism through secretion of BDNF targeting neuronal excitability with tropomyosin receptor kinase B (TrkB)^53^. Interestingly, microglia seem activated in a paracrine way through BDNF-TrkB signaling^54^. Moreover, neurotrophins such as BDNF and NGF can be retrogradely transported by axons^55^, a phenomenon amplified after peripheral nerve injury^56^ and may impact cognitive issues. Deciphering the kinetic and spatial/cellular repartition of neurotrophic factors after surgery may lead to implement new prognostic biomarkers, and better understand and prevent POCD in patients^57^.

In conclusion, our findings demonstrate for the first time that a muscle destruction alone can by itself lead to POCD occurrence. Microglial cell reactivity 24 h after surgery and late perturbations of brain neurotrophins BDNF and NGF seem to play a role in the pathophysiology. Our model can be a powerful tool to decipher influence of muscle tissue and microglia on cognitive decline occurrence after surgery, and to shed light on some misunderstood aspects of POCD pathophysiology.

## Data Availability

The data that support the findings of this study are available from the corresponding author upon reasonable request.
